# Multifunctionally disordered TiO_2_ nanoneedles prevent periprosthetic infection and enhance osteointegration by killing bacteria and modulating the osteoimmune microenvironment

**DOI:** 10.7150/thno.98219

**Published:** 2024-09-16

**Authors:** Yangmengfan Chen, Liqiang Zhou, Ming Guan, Shue Jin, Peng Tan, Xiaoxue Fu, Zongke Zhou

**Affiliations:** 1 Department of Orthopedics and Research Institute of Orthopedics, West China Hospital, Sichuan University, Chengdu, 610041, China.; 2 Department of Orthopedics, West China Hospital, Sichuan University, Chengdu, 610041, China.; 3 MOE Frontiers Science Center for Precision Oncology Faculty of Health Sciences, University of Macau, Macau SAR 999078, China.; 4 Department of Surgery, Beth Israel Deaconess Medical Center, Harvard Medical School, Boston, MA 02115, USA.; 5 Department of Orthopedic Surgery, The First Affiliated Hospital, Zhejiang University School of Medicine, Hangzhou 310003, China.

**Keywords:** titanium oxide, surface modification, periprosthetic infection, osteoimmunology, osteointegration

## Abstract

**Rationale:** Total hip arthroplasty (THA) and total knee arthroplasty (TKA) are effective interventions for end-stage osteoarthritis; however, periprosthetic infection is a devastating complication of arthroplasty. To safely prevent periprosthetic infection and enhance osteointegration, the surface modification strategy was utilized to kill bacteria, modulate the osteoimmune microenvironment, and improve new bone formation.

**Methods:** We used the hydrothermal method to fabricate a bionic insect wing with the disordered titanium dioxide nanoneedle (TNN) coating. The mussel-inspired poly-dopamine (PDA) and antibacterial silver nanoparticles (AgNPs) were coated on TNN, named AgNPs-PDA@TNN, to improve the biocompatibility and long-lasting bactericidal capacity. The physicochemical properties of the engineered specimen were evaluated with SEM, AFM, XPS spectrum, and water contact assay. The biocompatibility, bactericidal ability, and the effects on macrophages and osteogenic differentiation were assessed with RT-qPCR, Western blotting, live/dead staining, immunofluorescent staining, *etc*.

**Results:** The AgNPs-PDA@TNN were biocompatible with macrophages and exhibited immunomodulatory ability to promote M2 macrophage polarization. In addition, AgNPs-PDA@TNN ameliorated the cytotoxicity caused by AgNPs, promoted cell spreading, and increased osteogenesis and matrix deposition of BMSCs. Furthermore, AgNPs-PDA@TNN exhibited bactericidal ability against *E. coli* and *S. aureus* by the bionic nanostructure and coated AgNPs. Various imaging analyses indicated the enhanced bactericidal ability and improved new bone formation by AgNPs-PDA@TNN *in vivo*. H&E, Gram, and Masson staining, verified the improved bone formation, less inflammation, infection, and fibrosis encapsulation. The immunofluorescence staining confirmed the immunomodulatory ability of AgNPs-PDA@TNN *in vivo*.

**Conclusion:** The bionic insect wing AgNPs-PDA@TNN coating exhibited bactericidal property, immunomodulatory ability, and enhanced osteointegration. Thus, this multidimensional bionic implant surface holds promise as a novel strategy to prevent periprosthetic infection.

## 1. Introduction

Hip and knee arthroplasties are the most prevalent and efficacious interventional measures for end-stage osteoarthritis, with a survival rate exceeding 95% in a 10-year follow-up study 1, 2. Approximately one million total hip arthroplasty (THA) and total knee arthroplasty (TKA) procedures are carried out annually in the US, and this number is anticipated to increase persistently 3. However, the periprosthetic infection has the most severe complication, accounting for 14.8% and 25.2% of revisions after THA and TKA, respectively 4. It has been postulated that most periprosthetic infections occur during the operation or result from hematogenous infections 5. Hence, effective prevention and control strategies are essential.

Biocompatibility is one of the most fundamental characteristics in the field of biomaterials. The term “biocompatibility” could be interpreted as the implant's ability to safely perform the desired biological function and elicit an appropriate host response during treatment 6. Although the FDA has approved titanium (Ti) for medical applications, its biocompatibility is insufficient. The Ti implant might lead to fibrous encapsulation through a complex biological response, eventually leading to implant failure 7. Nevertheless, with the development of the interdisciplinary fields of medical sciences and engineering, surface modification emerged as an established technique for actively carrying out therapeutic tasks and avoiding side effects, *e.g.*, preventing infection, promoting osteogenesis, regulating immune response, and avoiding fibrous encapsulation, thereby improving the biocompatibility of implant materials 8.

Based on coating preparation approaches, surface modification is classified into chemical, physical, or composite modification 9. The appropriate surface modification demonstrates outstanding performance in regulating cellular activities. For instance, the plasma surface modification can create a hydrophilic surface with enhanced cell adhesion 10. The zwitterionic surface modification is characterized by positive and negative charges, thereby preventing foreign body reactions 11. Morphology modifications of nano-surfaces significantly impact the binding, conformation, and activities of plasma proteins 12. Consequently, the modified surface morphology can alter the cytoskeleton, cellular activities, and differentiation 13.

With the advances in nanotechnology and biomedicine, TiO_2_-based nanomaterials have been widely used due to their low cytotoxicity, nanostructure, and excellent biocompatibility 14. The nanotube is an early biomimetic TiO_2_ coating, which demonstrated antimicrobial activity and improved biocompatibility, owing to the intimate connection between morphology and biological effects 15. Recently, with the development of synthesis techniques and precise adjustment of surface parameters, the surface morphology and mechanical properties of TiO_2_-based nanomaterials have been changed. Examples include the fabrication of nanodots 16, nanopillar 17, and nanoneedles. Salatto *et al.* synthesized the vertically oriented polystyrene-block-poly(methyl methacrylate) cylindrical nanostructure and coated it on TiO_2_ nanopillars. Their study demonstrated that the end edge of the oriented TiO_2_ nanostructure could absorb the lipid heads and modify the shape of a lipid bilayer, leading to the high force stress of the bacterial membrane and ultimately killing bacteria 18. There are three distinct deformations of bacterial cell walls due to the nanostructure-induced mechanical stress resulting in outward, inward, and flat deformation in the bound region. Furthermore, the surface with sharp nanopillars results in a higher intrinsic pillar pressure per square area, thus presenting a stronger bactericidal ability 19.

Considering these deformation features of bacterial cell membranes and inspired by the nanostructure of insect wings, we fabricated the disordered titanium dioxide nanoneedle (TNN) coating on the surface of pure-Ti using the hydrothermal method. The advantage of the disordered TNN lies in the improved mechanical property and the sharp tip. More importantly, the disordered arrangement of TNN facilitates increased outward deformation and avoids flat deformation thus leading to stronger bactericidal ability than ordered and vertically oriented nanostructures. In our previous study, we fabricated the phytic acid - Zn^2+^ coordinated TiO_2_ nanopillar arrays and oxygen self-supporting CaO_2_ nanoparticles, which exhibited mechano-bactericidal effects against nascent biofilms and neutrophil-modulatory ability 20. Silver nanoparticles (AgNPs) were deposited on the surface of TNN (AgNPs@TNN) to enhance their anti-planktonic ability further.

AgNPs have been widely acknowledged as a broad-spectrum bactericidal agent and utilized in medical products and daily life 21. It has been reported that AgNPs kill bacteria in multiple ways, like disrupting the bacterial cell wall, denaturing respiratory enzymes, ROS production, and impairing ribosomes and DNA 22. However, the bactericidal ability of AgNPs is a double-edged sword. The large burst release of AgNPs might also harm the host cells and tissues, thereby impeding the healing process 23. Therefore, an effective release control strategy is crucial for the safety and therapeutic effects of implant coatings. We addressed the release control challenge by using poly-dopamine (PDA) to fabricate PDA@TNN and AgNPs-PDA@TNN. PDA is inspired by the foot protein of mussels and exhibits minimal cytotoxicity, favorable biodegradability, and adhesive properties, and is hence adopted to facilitate drug loading and release control 24. In recent years, the antioxidant capacity of PDA has been elucidated, which can effectively scavenge ROS and reduce inflammation 25. Therefore, incorporating PDA in this study alleviated oxidative stress and inflammation and offered a sustained release of Ag^+^.

Osteoimmunology has recently attracted significant attention in bone healing 26. The cross-talk between bone marrow mesenchymal stem cells (BMSCs) and immune cells within the microenvironment plays a crucial role in modulating the homeostasis and regeneration of bone 27. Macrophages are one of the most representative cell types in the osteoimmune microenvironment due to their diverse phenotype and specialized function 28. Macrophages are typically classified into two subsets: pro-inflammatory (M1) and anti-inflammatory (M2) 29. M1 macrophages can synthesize and release a variety of inflammatory cytokines, including interferon-γ (IFN-γ), tumor necrosis factor-α (TNF-α), and granulocyte-macrophage colony-stimulating factor (GM-CSF), thereby causing excessive inflammation and tissue damage 30. In contrast, M2 macrophages are widely recognized for their outstanding ability in tissue remodeling and repair 31. Therefore, most orthopedic implants were designed to modulate the polarization of macrophages towards the M2 phenotype, which can alleviate inflammation, promote angiogenesis, and enhance bone regeneration and osteointegration 32. The surface topography at the nanoscale has been extensively reported to have remarkable influences on cell proliferation and differentiation 33. The shape and size of the nanostructure can effectively alter the phenotype and functionality of macrophages 34. Nevertheless, the immunomodulatory ability of TNN on macrophage polarization remains unclear.

In this study, we exploited the rational combination of the disordered TNN, PDA, and AgNPs, leveraging the advantages of each component. AgNPs-PDA@TNN exhibited great potential as a novel and effective implant coating for eliminating osteomyelitis, modulating the osteoimmune microenvironment, and enhancing osteointegration.

## 2. Results

### 2.1 Characterization of disordered TiO_2_ nanoneedle coating

**Figure 1:**
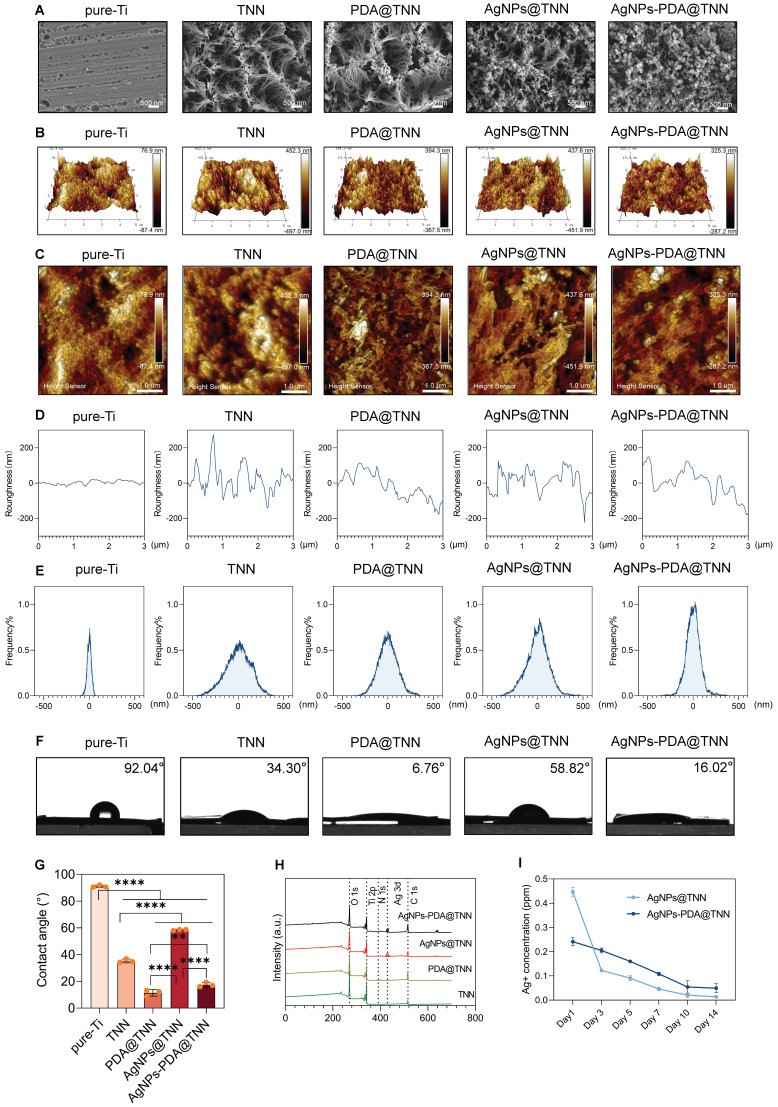
** Surface characterization of samples. (A)** SEM scanning images of the morphology of disordered TNN coating, PDA layer, and AgNPs **(B)** 3D images and **(C)** 2D images of AMF indicating the surface roughness of each sample **(D)** Surface roughness and** (E)** Frequency distributions of the roughness of each specimen were quantified**. (F)** Hydrophilicity was evaluated by the water contact assay, **(G)** Water contact angles of each specimen were quantified, indicating that the TNN surface and PDA coating decreased but the loading of AgNPs increased the water contact angle.** (H)** XPS spectrum verified the coating of PDA and/or AgNPs on the sample surface.** (I)** Release curve of Ag^+^ demonstrated that AgNPs@TNN led to a burst release of Ag^+^, while AgNPs-PDA@TNN exhibited a slow and steady release of Ag^+^. N ≥ 3, n = 3. Data were compared by non-parametric one-way ANOVA followed by Tukey's multiple comparison test: **p < 0.01, and ****p < 0.0001.

The surface topography of pure-Ti, TiO_2_ nanoneedles (TNN), poly-dopamine-coated TNN (PDA@TNN), silver nanoparticles-coated TNN (AgNPs@TNN), and AgNPs-loaded PDA@TNN (AgNPs-PDA@TNN) was illustrated by scanning electron microscopy (SEM) **(Figure 1A)**. The surface of the TNN coating exhibited topographic characteristics similar to the nanoprotrusions on insect wings, such as cicada and dragonfly wings 35. This ingenious surface topography has achieved outstanding biological properties through natural evolution and selection 36. The surface roughness was evaluated using atomic force microscopy (AFM) to acquire insights into the physiochemical features of the coatings. The 3D and 2D topographies of each specimen are presented in **Figures 1B** and **1C**. Furthermore, the surface roughness **(Figure 1D)** and the roughness distribution frequency **(Figure 1E)** were quantified. Overall, the comprehensive results of AFM indicated that TNN presented greater surface roughness than pure-Ti, and the PDA coating slightly diminished the surface roughness of TNN.

Hydrophilicity is a crucial factor of implant materials influencing biocompatibility. Also, the hydrophobic surface of implants leads to stronger protein adsorption, inflammation, and foreign body reaction, thereby hindering the functionality and longevity of the implants 37. We assessed surface hydrophilicity was assessed by using the water contact angle assay and observed. a water contact angle of 92.04° for pure Ti, while the morphological coating of TNN exhibited a water contact angle of 34.30°. The hydrophilic property of the bio-inspired PDA coating further decreased the water contact angle of PDA@TNN to 6.76°. In contrast, the loading of AgNPs increased the water contact angle to 58.82°. To balance the hydrophilicity and functionality, AgNPs-PDA@TNN presented a compromised water contact angle of 16.02° **(Figure 1F-G)**. The X-ray photoelectron spectroscopy (XPS) spectrum verified the successful coating of PDA and/or AgNPs on the surface of the substrate **(Figure 1H)**.

AgNP is a widely utilized inorganic bactericidal agent that can effectively disrupt biofilm and kill bacteria by contact-killing 38 and ROS production 39. However, the burst release of AgNPs or Ag^+^ is cytotoxic to host tissues and cells 40. For instance, the excess accumulation of AgNPs may lead to oxidative stress 41, inflammation 42, DNA damage 43, lysosome damage 44, and mitochondria-mediated apoptosis 45. As is evident from the release curve of Ag^+^, AgNPs@TNN led to a burst release of Ag^+^ in one day, which is detrimental to the healing process. In contrast, AgNPs-PDA@TNN demonstrated a slow and steady release of Ag^+^ due to the mucoadhesive coating of PDA, thus providing a balanced bactericidal ability and biosafety of the implant **(Figure 1I)**.

### 2.2 Biocompatibility of disordered TiO_2_ nanoneedle coating with macrophages

**Figure 2:**
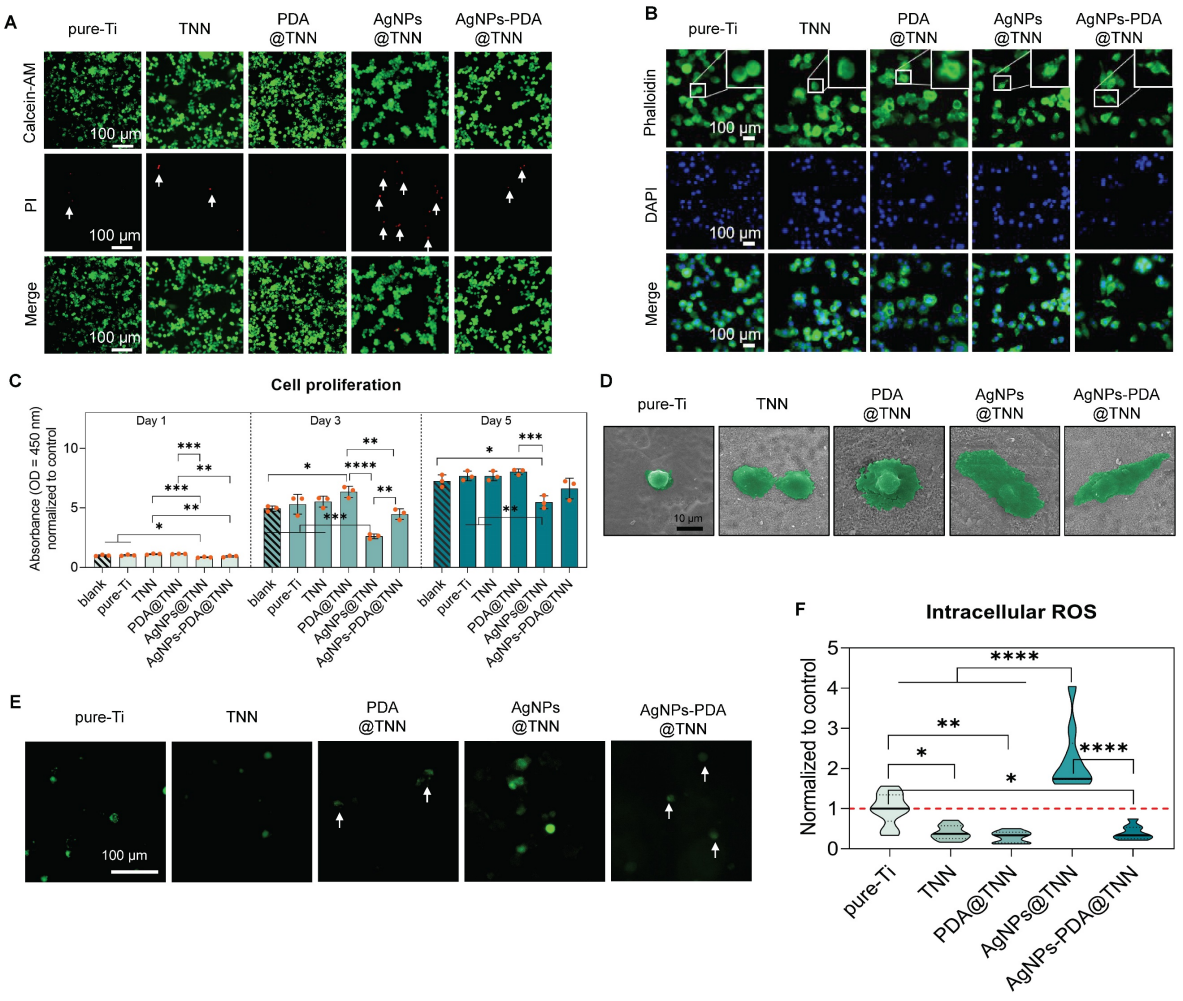
** Biocompatibility of samples with macrophages. (A)** Live/dead staining to evaluate the cytotoxicity of samples **(B)** Cytoskeletal staining to investigate the influence of samples on macrophage shape change **(C)** Cell proliferation of adhered macrophages on samples on day days 1, day 3, and day5 were evaluated by the CCK-8 assay **(D)** SEM images of macrophages on the surface of samples **(E, F)** Representative images and the quantitative results of intracellular ROS levels in macrophages visualized by the DCFH-DA probe. The red dashed line refers to the normalized basal level of control. N ≥ 3, n = 3. Data were compared by non-parametric one-way ANOVA followed by Tukey's multiple comparison test: * p < 0.05, ** p < 0.01, *** p < 0.001, and **** p < 0.0001.

Live/dead staining was conducted to discriminate between the live and dead cells resulting from the cytotoxicity of the sample. Few dead cells (indicated by white arrows) were observed on pure-Ti and TNN due to the normal level of apoptosis. PDA is a biocompatible polymer utilized to improve the biocompatibility of implants 14. In this study, no dead cells were detected on the surface of PDA@TNN. In contrast, AgNPs@TNN induced cell death of macrophages due to the burst release of Ag^+^. However, AgNPs-PDA@TNN circumvented the cellular damage caused by the burst release of Ag^+^ due to the PDA coating** (Figure 2A)**. The topographical property of the implant surface has been reported to regulate cytoskeleton remodeling and modify the morphology of macrophages 15. As the cytoskeletal staining showed, the adhered macrophages exhibited a round shape on pure-Ti, suggesting a non-activated state. Macrophages on the surface of TNN and PDA@TNN tended to generate broad pseudopodia. In contrast, macrophages on the surface of AgNPs@TNN and AgNPs-PDA@TNN exhibited a spindle-like shape **(Figure 2B)**.

Macrophage proliferation was determined with the CCK-8 assay to quantify the biocompatibility of each sample further. Compared with the blank group and pure-Ti, AgNPs@TNN exhibited a significant decrease in macrophage proliferation on days 1, 3, and 5. On the contrary, the macrophage proliferation was not significantly impaired on the surface of AgNPs-PDA@TNN due to the elimination of the Ag^+^ burst release, indicating a reduced cytotoxicity of AgNPs-PDA@TNN **(Figure 2C)**. The morphological alterations of the adhered macrophages on each sample were verified by SEM **(Figure 2D)** and were found to be consistent with cytoskeletal staining. The intracellular ROS level was evaluated with the DCFH-DA assay to explore the mechanism underlying the biological effects. The results indicated that the cytotoxicity of AgNPs@TNN might be due to excessive ROS accumulation. In contrast, PDA might contribute to the ROS scavenging ability resulting in improved biocompatibility **(Figure 2E)**. Moreover, the quantitative results verified the increased ROS generation in macrophages on the surface of AgNPs@TNN **(Figure 2F)**.

### 2.3 Influence of disordered TiO_2_ nanoneedle coating on macrophage polarization

**Figure 3:**
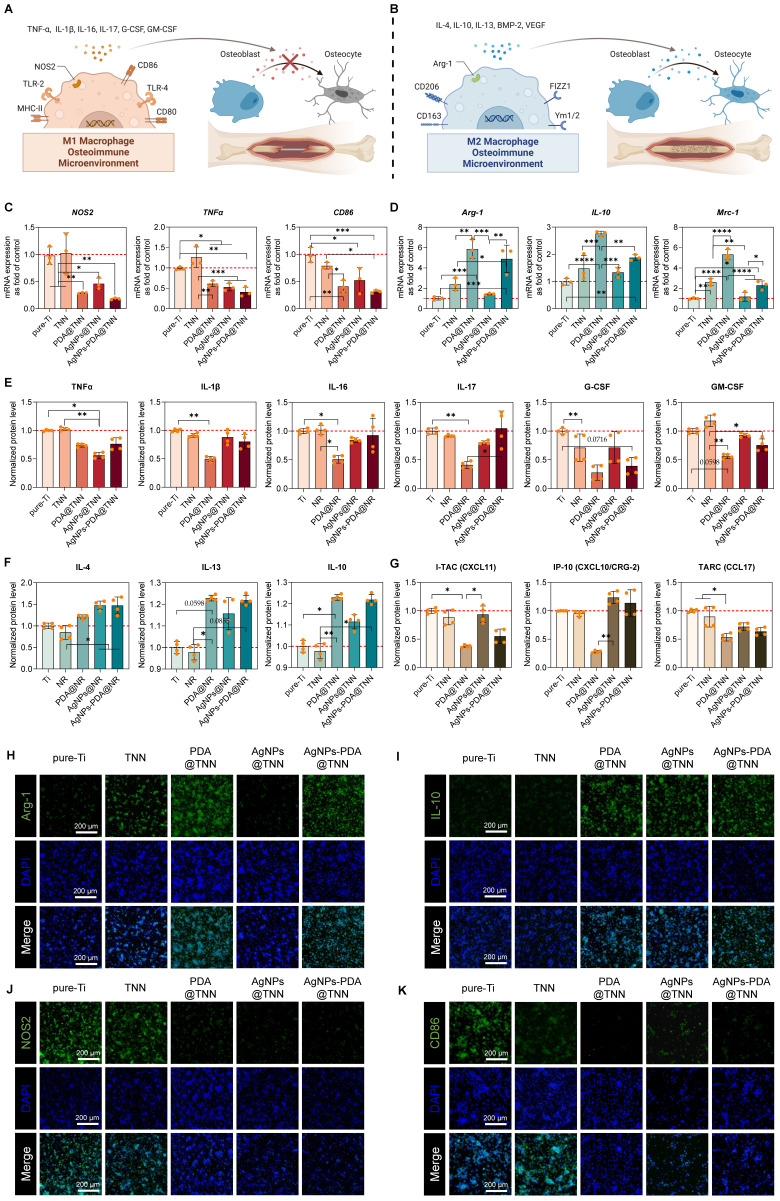
** Effect of samples on macrophage polarization.** Illustrative scheme of **(A)** M1 and **(B)** M2 macrophages in the osteoimmune microenvironment. Gene expression of** (C)** M1 macrophage markers *(NOS2*, *TNFα*, and *CD86*), and **(D)** M2 macrophage markers (Arg*-1*, *IL-10,* and *Mrc-1*). Protein secretion of **(E)** Pro-inflammatory cytokines (TNFα, IL-1β, IL-16, IL-17, G-CSF, and GM-CSF), **(F)** Anti-inflammatory cytokines (IL-4, IL-10, and IL-13), and **(G)** chemokines (CXCL-10/CRG-2, CXCL-11, and CCL-17). Immunofluorescence staining of **(H)** Arg-1, **(I)** IL-10**, (J)** NOS2, and** (K)** CD86. The red dashed line refers to the normalized basal level of control. N ≥ 3, n = 3. Data were compared by non-parametric one-way ANOVA followed by Tukey's multiple comparison test: * p < 0.05, ** p < 0.01, *** p < 0.001, **** p < 0.0001.

Macrophages exhibit functional plasticity in the osteoimmune microenvironment. The inactivated macrophage (M0) can polarize into either pro-inflammatory macrophage (M1) **(Figure 3A)** or pro-healing macrophage (M2) **(Figure 3B)**, subsequently influencing bone healing 46. In this study, we performed a comprehensive evaluation of the M1 (NOS2, TNFα, IL-1β, IL-16, *etc.*) and M2 markers (Il-4, IL-10, IL-13, *etc.*) and several chemokines of adhered macrophages on the surface of each sample.

Cytokines secreted by macrophages are crucial for the immune microenvironment. For instance, nitric oxide synthase 2 (NOS2) leads to the generation of excessive nitric oxide (NO) and reactive oxygen species (ROS), initiating the signaling pathways to facilitate M1 macrophage polarization with bactericidal ability 47. TNFα, IL-1β, IL-16, and IL-17 are the typical pro-inflammatory cytokines that exacerbate inflammation, compromise the bone healing process, and are considered M1 macrophage markers 48. CD86 is also considered a surface marker of M1 macrophages 49. Conversely, IL-4, IL-10, and IL-13 are typical anti-inflammatory cytokines defined as M2 markers 50. Arg-1, a cytosolic enzyme and the substrate of NOS2, can also exhibit anti-inflammatory ability and is, therefore, regarded as an M2 macrophage maker 51. MRC-1, also known as CD206, is a transmembrane glycoprotein and is widely defined as an M2 macrophage marker 52. GM-CSF and G-CSF, members of the CSF family, reflect the inflammatory levels and also impact bone healing 53. Moreover, CXCL-10/CRG-2, CXCL-11, and CCL-17 play important roles in inflammation and regulate the secretion of inflammatory cytokines, angiogenesis, and tissue regeneration 54.

We observed downregulation of *NOS2*, *TNFα*, and *CD86,* indicative of M1 macrophage phenotype, *e.g.*, on PDA@TNN, AgNPs@TNN, and AgNPs-PDA@TNN **(Figure 3C)**. In contrast, M2 macrophage markers *Arg-1*, *IL-10*, *Mrc-1,* were up-regulated on TNN, PDA@TNN, and AgNPs-PDA@TNN **(Figure 3D)**. The macrophage polarization was further verified by protein secretion using a cytokine array. The results demonstrated that the secretion of several pro-inflammatory cytokines, *e.g.*, TNFα, IL-1β, IL-16, IL-17, G-CSF, and GM-CSF, decreased on PDA@TNN, suggesting that PDA plays the key role in reducing M1 macrophage polarization **(Figure 3E)**. On the contrary, secretion of anti-inflammatory cytokines IL-4, IL-10, and IL-13, was highly increased on PDA@TNN, AgNPs@TNN, and AgNPs-PDA@TNN **(Figure 3F)**. Moreover, the secretion of chemokines, *e.g.*, CXCL-10/CRG-2, CXCL-11, and CCL-17, was reduced in the presence of PDA coating **(Figure 3G)**.

We also conducted immunofluorescence staining to verify the immunomodulatory ability of each specimen. The results indicated that the fluorescence intensities of Arg-1 **(Figure 3H)** and IL-10 **(Figure 3I)** were high on PDA@TNN and AgNPs-PDA@TNN, and of NOS2 **(Figure 3J)** and CD86 **(Figure 3K)** were low on PDA@TNN, AgNPs@TNN, and AgNPs-PDA@TNN. These results demonstrated that the morphology of TNN and the introduction of PDA facilitated M2 macrophage polarization in the immune microenvironment.

### 2.4 TiO_2_ disordered nanoneedle coating promotes the spreading and osteogenesis of BMSCs

The cell behavior can be modulated by the surface properties of biomaterials, such as physical characteristics (topography, roughness, and stiffness) and surface chemistry (charge and wettability) 8. For instance, the surface topography of nanowire scaffolds possesses elastic focal points, facilitating MSC adhesion and promoting osteogenic differentiation 55. The specific nanostructure of the surface alters the cellular contact and cytoskeletal polymerization of BMSCs, eventually modulating osteogenic differentiation 56. Mechanical signals of different materials can also direct cell differentiation 57. TiO_2_ nanotubes have recently been reported to have the ability to activate mitochondrial oxidative phosphorylation through the mechanosensitive calcium channel Piezo1, resulting in enhanced osteogenesis with increased Ca^2+^ influx 58. In addition to nanotopography, different surface modification strategies can actively modulate cell behavior. PDA contains hydroxyl and amino groups and can improve the hydrophilicity of the surface 59. Therefore, the PDA-modified surface can promote cell adhesion 60 and reduce oxidative stress 61 and foreign body reaction 8. At the molecular level, it is widely acknowledged that the surface properties of biomaterials can modulate a variety of metabolic pathways, including mitochondrial activities 62, autophagy 63, energy metabolism 64, and other complex metabolic networks.

**Figure 4:**
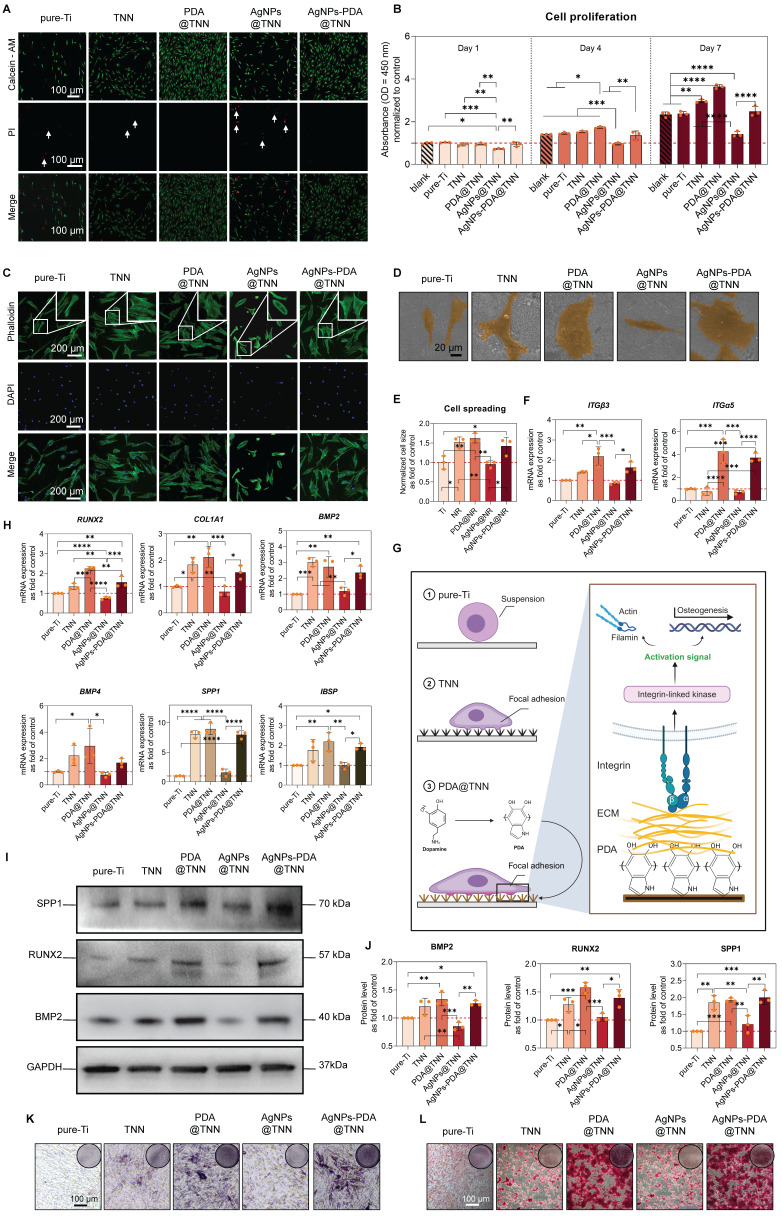
** Cell viability, spreading, and osteogenesis of BMSCs on each sample. (A)** Live/dead staining to evaluate the cytotoxicity of each sample on adhered BMSCs. **(B)** Cell proliferation of adhered BMSCs on each sample on days 1, 4, and 7 by the CCK-8 assay. **(C)** Cytoskeletal staining to investigate the spreading of BMSCs. **(D)** SEM images of BMSCs on the surface of each sample **(E)** Quantitative results of cell spreading. **(F)** Gene expression of *ITGβ3* and *ITGα5 in* adhered BMSCs **(G)** Illustrative scheme depicting the importance of *ITGβ3* and *ITGα5* in cell adhesion. **(H)** Gene expression of osteogenic markers in BMSCs in the co-culture model.** (I)** Western blot images **(J)** Quantitative results of BMP2, RUNX2, and SPP1 protein levels. **(K)** ALP staining to evaluate the early osteogenic differentiation of BMSCs on day 14. **(L)** Alizarin red staining to evaluate the mineral deposition on day 28. The red dashed line refers to the normalized basal level of control. N ≥ 3, n = 3. Data were compared by non-parametric one-way ANOVA followed by Tukey's multiple comparison test: * p < 0.05, ** p < 0.01, *** p < 0.001, **** p < 0.0001.

Considering the close relationship between surface properties and osteogenesis, we evaluated the BMSC behavior of each sample in this study. The live/dead staining of BMSCs was conducted to assess the biocompatibility of each sample. A few dead cells were observed on the surface of pure-Ti and TNN. In contrast, the burst release of Ag^+^ from AgNPs@TNN led to an increased number of dead cells. The PDA coating converted the burst release into a controlled release, decreasing the number of dead cells on the surface of AgNPs-PDA@TNN **(Figure 4A)**. Cell proliferation was also evaluated using the CCK-8 assay. The results demonstrated excellent biocompatibility of TNN, PDA@TNN, and AgNPs-PDA@TNN. However, the proliferation of BMSCs on the surface of AgNPs@TNN was significantly reduced due to the burst release of Ag^+^ from AgNPs@TNN **(Figure 4B)**.

The attachment and spreading of BMSCs are widely recognized as critical factors for osteogenic differentiation 65. Cytoskeletal staining was performed to evaluate the morphology of BMSCs on each sample. As the representative images showed, BMSCs exhibited an elongated shape and lamellipodia on pure-Ti caused by the physiochemical properties of the surface, such as low roughness and high hydrophilicity. BMSCs showed an elliptical morphology due to the low biocompatibility of the AgNPs@TNN. On the contrary, the surface of TNN, PDA@TNN, and AgNPs-PDA@TNN elicited cytoskeleton remodeling and improved the spreading of BMSCs **(Figure 4C)**. The morphological alternations were further verified by SEM images **(Figure 4D)**. The level of cell spreading of BMSCs on each sample was quantified by evaluating the cytoplasmic to nuclear ratio 66. The quantitative results verified that TNN, PDA@TNN, and AgNPs-PDA@TNN significantly increased cell spreading **(Figure 4D)**.

The molecular mechanism of cell adhesion was investigated by evaluating *integrin* (*ITG*) gene expression, which is important for cell adhesion. The results indicated that PDA@TNN and AgNPs-PDA@TNN significantly up-regulated the gene expression of *ITGβ3* and *ITGα5*, probably due to the presence of PDA coating **(Figure 4F and 4G)**. The osteogenesis of BMSCs in the osteoimmune microenvironment was evaluated using the indirect co-culture model. The conditioned medium from TNN, PDA@TNN, and AgNPs-PDA@TNN significantly up-regulated the gene expression of *RUNX2, COL1A1, BMP2, BMP4, SPP1*, and *IBSP* **(Figure 4F)**. In addition, the protein levels of BMP2, RUNX2, and SPP1 by Western blotting verified the positive effects of TNN, PDA@TNN, and AgNPs-PDA@TNN on osteogenesis **(Figures 4I and 4J)**. Lastly, the ALP staining **(Figure 4K)** performed on day 14 and alizarin red staining **(Figure 4L)** on day 28 indicated improved osteogenic differentiation and mineral deposition of BMSCs in the TNN, PDA@TNN, and AgNPs-PDA@TNN groups.

### 2.5 Bactericidal ability of disordered TiO_2_ nanoneedle coating

**Figure 5:**
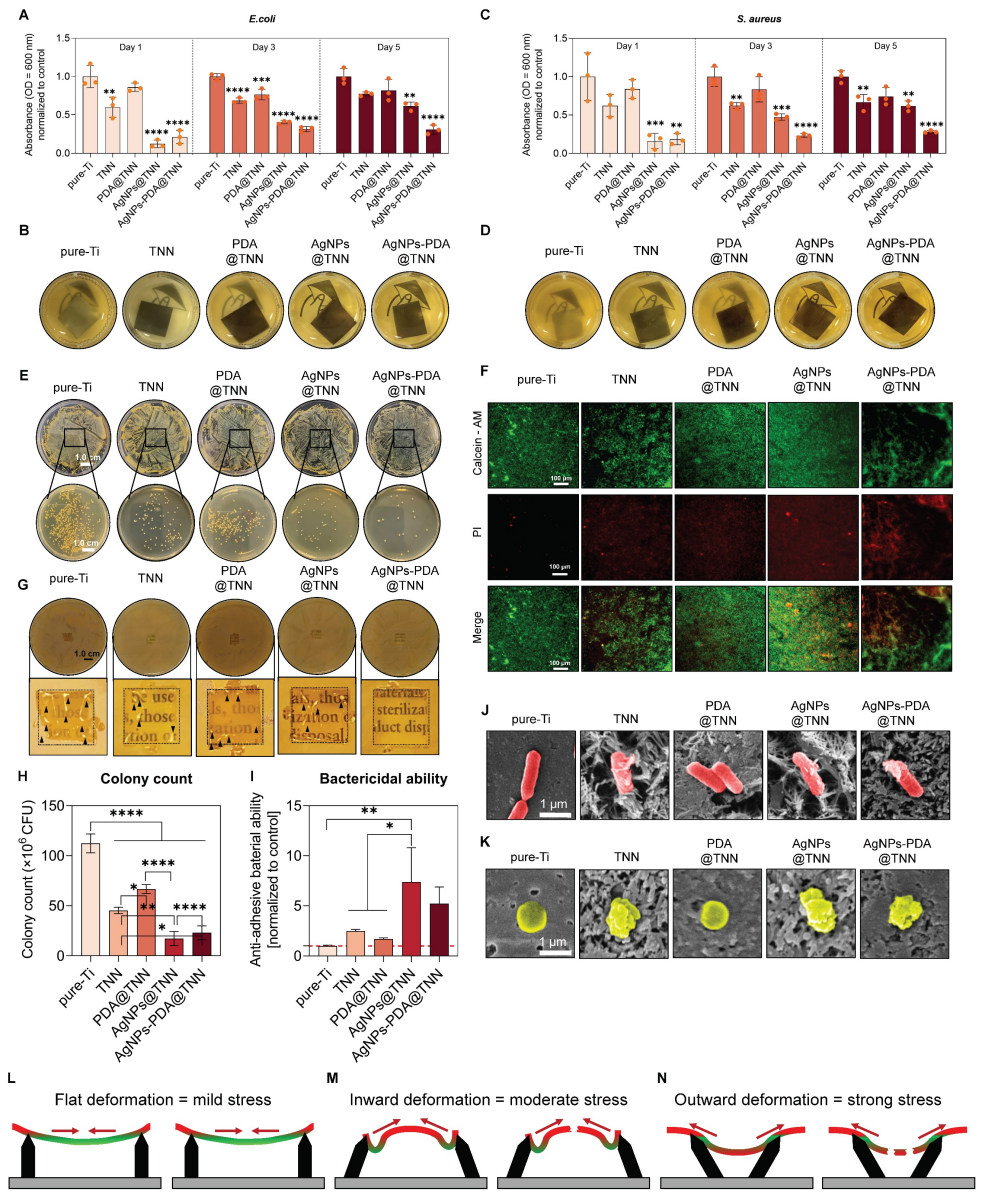
** Bactericidal ability against adherent bacteria, planktonic bacteria, and biofilm. (A)** Proliferation of *E. coli* on days 1, 3, and 5 was measured at OD 600 nm. **(B)** Gross images of the culture medium of *E. coli*. **(C)** Proliferation of *S. aureus* on days 1, 3, and 5 was measured at OD 600 nm. **(D)** Gross images of the culture medium of *S. aureus*. **(E)** Bacteria were cultured on the agar plate to evaluate the ability of anti-planktonic bacteria. **(F)** Live/dead staining to evaluate the bactericidal ability of each sample against adhesive bacteria. **(G)** Anti-biofilm assay to evaluate the anti-biofilm ability of each sample. Quantitative results of **(H)** Colony count of adhesive bacteria and **(I)** Bactericidal ability of each sample against the adhesive bacteria. Representative SEM images of **(J)** *E. coli* and** (K)** *S. aureus* on each sample **(L-N)** Illustrative scheme of the mechanobactericidal ability of vertical TNN and disordered TNN. The red dashed line refers to the normalized basal level of control. N ≥ 3, n = 3. Data were compared by non-parametric one-way ANOVA followed by Tukey's multiple comparison test: * p < 0.05, ** p < 0.01, *** p < 0.001, **** p < 0.0001.

Periprosthetic infections are the most severe complication in orthopedic surgery 67. The traditional orthopedic implants, *i.e.*, Ti and Ti alloy, lack antibacterial capacities, increasing the risk potential of bacterial adhesion and resulting in implant failure 68. The long-term bactericidal efficacy against planktonic bacteria was evaluated by measuring the proliferation of *E. coli* at 600 nm OD on days 1, 3, and 5 **(Figure 5A)**. As the gross images indicated, the bacterial proliferation was significantly restrained in the AgNPs@TNN and AgNPs-PDA@TNN groups **(Figure 5B)**. Similarly, the proliferation of *S. aureus* was also significantly limited in the presence of AgNPs **(Figure 5C and 5D)**. The bacterial suspension was collected and seeded on agar plates at different dilutions to evaluate the bactericidal ability. As displayed in **Figure 5E**, TNN, AgNPs@TNN, and AgNPs-PDA@TNN exhibited excellent anti-bacterial ability against planktonic bacteria **()**.

The bactericidal ability against adherent bacteria was further evaluated through live/dead staining. There were few dead bacteria on the surface of pure-Ti. In contrast, TNN, PDA@TNN, AgNPs@TNN, and AgNPs-PDA@TNN exhibited strong anti-bacterial ability **(Figure 5F)**. The anti-biofilm ability was evaluated by inversely placing each sample on the agar plate seeded with bacteria. The results revealed the poor anti-biofilm ability of pure-Ti with the presence of bacterial colonies (indicated by black arrows). PDA@TNN also exhibited weak anti-biofilm ability as the PDA coating covered the nanoneedle morphology. In contrast, TNN, AgNPs@TNN, and AgNPs-PDA@TNN exhibited strong bactericidal ability with few bacterial colonies and improved visibility of the agar gel, demonstrating complete eradication of the biofilm on the agar plate **(Figure 5G)**. Moreover, the bactericidal ability of each sample against the adherent bacteria was quantified, indicating that the number of bacterial colonies on the surface of TNN, AgNPs@TNN, PDA@TNN, and AgNPs-PDA@TNN was significantly reduced compared with pure-Ti **(Figure 5H)**. The bactericidal ability of AgNPs@TNN was significantly enhanced compared with pure-Ti, and the bactericidal ability of AgNPs-PDA@TNN was also potent but without statistical significance **(Figure 5I)**.

SEM was utilized to observe the morphological changes of *E. coli* **(Figure 5J)** and *S. aureus* **(Figure 5K)**. As the representative SEM images showed, *E. coli* and *S. aureus* were punctured by the nanoneedles on the surface of TNN, leading to the leakage of the cellular contents. The mechanobactericidal ability of nanostructure was caused by the flat, inward, and outward deformations of the bacterial cell wall. As displayed in the schematic, the disordered TNN demonstrated stronger mechanobactericidal ability than vertical TNN due to its ability to induce inward and outward deformation. In summary, the disordered TNN exhibited an excellent mechanobactericidal ability, and the PDA and AgNP coatings further endowed it with the anti-planktonic and long-lasting bactericidal capacity.

### 2.6 Disordered TiO_2_ nanoneedle coating prevents periprosthetic infection and promotes osteointegration *in vivo*

**Figure 6:**
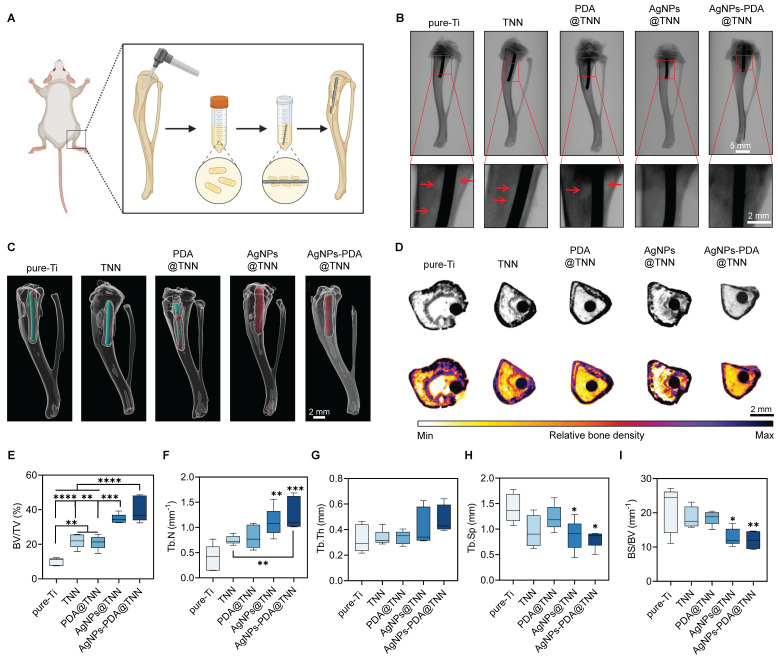
** Tibial osteomyelitis model in SD rats (A)** Illustrative scheme of the surgical procedure **(B)** X-ray images of the tibia. **(C)** 3D reconstruction of the tibia to visualize the newly formed bone around the implant. **(D)** Micro-scanning images of the tibia. Dead and reactive bones are presented in a pseudocolor visualization. Quantitative analysis of **(E)** Ratio of bone volume to total volume, **(F)** Number of trabecular bones, **(G)** Thickness of trabecular bones, **(H)** Spacing of trabecular bones, and **(I)** Ratio of bone surface to bone volume. N ≥ 3, n = 3. Data were compared by non-parametric one-way ANOVA followed by Tukey's multiple comparison test: * p < 0.05, ** p < 0.01, *** p < 0.001, **** p < 0.0001.

Periprosthetic infection is a disastrous complication following bone surgery and poses a severe threat to health 69. The adhesive bacteria on the implant surface lead to the formation of biofilm, which is widely regarded as the primary cause of periprosthetic infection 70. This studyused bacteria-immersed implants to establish the tibial osteomyelitis model in SD rats (**Figure 6A**). As demonstrated by the X-ray images, extensive periprosthetic osteolysis (indicated by the red arrows) was observed around pureTi. However, the nanoneedle surface reduced the osteolysis area around TNN and PDA@TNN. In contrast, no osteolysis was detected around AgNPs@TNN and AgNPs-PDA@TNN due to the release of Ag^+^ (**Figure 6B**). The tibia 3D reconstruction was performed to evaluate the bone healing around the implant. Representative images verified the different abilities of specimens in preventing periprosthetic osteolysis. The surfaces of AgNPs@TNN and AgNPs-PDA@TNN were extensively covered by the newly formed bone instead of osteolysis (**Figure 6C**).

Furthermore, micro-CT was used to assess the infection and bone regeneration. As is evident by the CT scanning, the reactive bone and dead bone formed a bone shell around Ti, while the infection area was diminished around TNN due to the surface topography **(Figure 6D)**. Bone regeneration was quantified to assess the therapeutic effects of TiO_2_ nanoneedle coating. The ratio of BV/TV indicated that TNN and PDA@TNN increased the bone volume, which was further enhanced by AgNPs@TNN and AgNPs-PDA@TNN due to their bactericidal ability **(Figure 6E)**. The analysis of bone microarchitecture verified the increased number **(Figure 6F)** and thickness **(Figure 6G)** of trabecular bone and decreased spacing **(Figure 6H)** at the non-infectious bone marrow cavity. Moreover, the ratio of bone surface to bone volume verified increased osteointegration by AgNPs@TNN and AgNPs-PDA@TNN **(Figure 6I)**.

### 2.7 Histological analysis of the therapeutic effects of TiO_2_ nanoneedle coating *in vivo*

**Figure 7:**
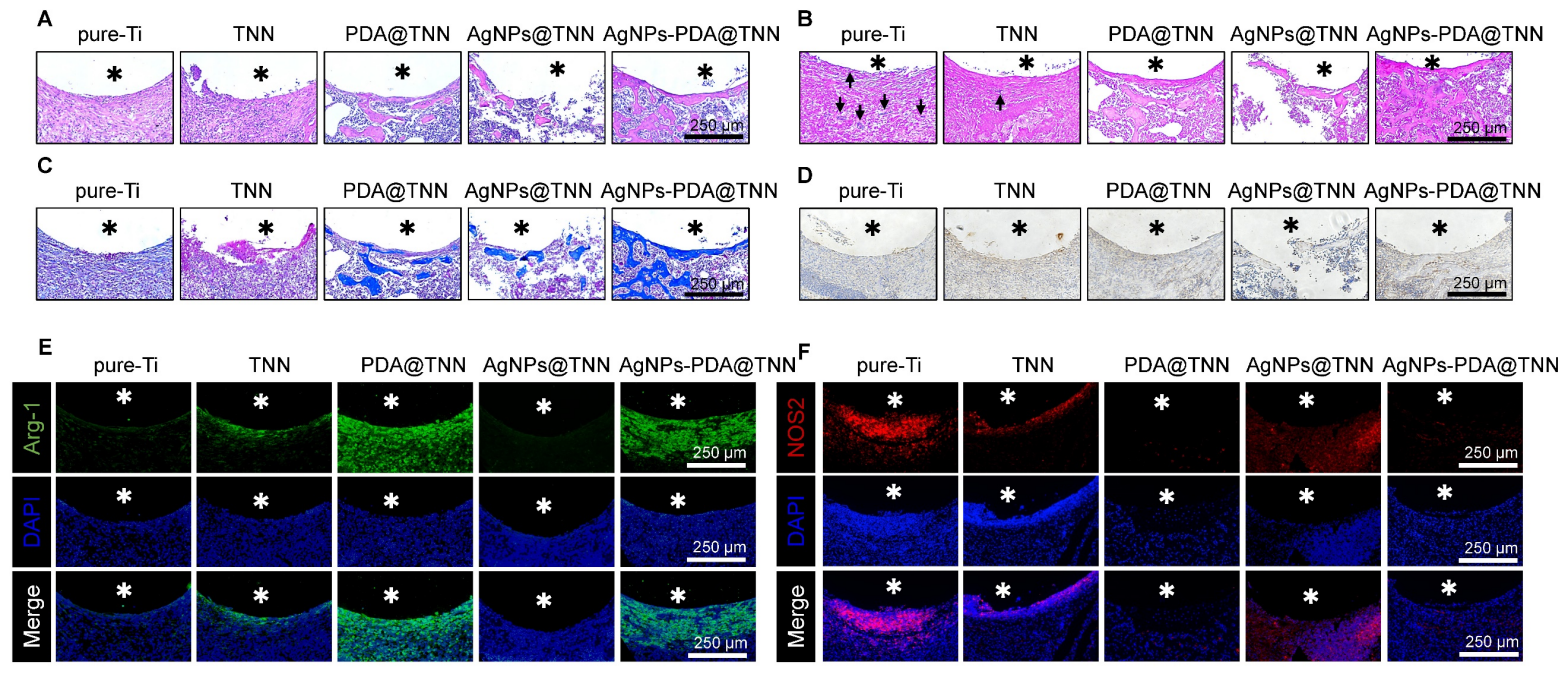
** Histological analyses of the therapeutic effects *in vivo*. (A)** H&E staining to observe immune cell infiltration and new bone formation. **(B)** Gram staining to assess the infection. **(C)** Masson staining to evaluate the level of fibrosis. **(D)** Immunohistochemical staining of BMP-2. Immunofluorescence staining of **(E)** Arg-1 and **(F)** iNOS was conducted to evaluate the alternation of the immune microenvironment. Asterisks represent implant areas.

Histological analysis was performed to evaluate the therapeutic efficacy of TiO_2_ nanoneedle coating on tibial osteomyelitis. H&E staining revealed minimal bone formation around Ti. Nevertheless, the AgNPs@TNN group indicated a reduced population of tissue cells, disruption of structural integrity surrounding the implant, and impaired osteointegration, induced by the burst release of Ag^+^ from AgNPs@TNN, leading to cell damage, apoptosis, and tissue destruction. In contrast, AgNPs-PDA@TNN exhibited negligible tissue destruction and enhanced osteointegration around the implant **(Figure 7A)**. Gram staining was used to visualize and discriminate between Gram-positive and Gram-negative bacteria 71. The results indicated the presence of bacteria around Ti (marked with black arrows), whereas the infection was alleviated around TNN and even eradicated in the presence of AgNPs **(Figure 7B)**. Mason staining showed a fibrotic encapsulation of pure-Ti indicative of a foreign body reaction 8. However, the TNN surface could mitigate the foreign body reaction and improve biocompatibility **(Figure 7C)**. Furthermore, immunohistochemical staining conducted to evaluate the BMP-2 level suggested that TNN and AgNPs-PDA@TNN strongly increased the secretion of BMP-2 around the implant **(Figure 7D)**. Immunofluorescence staining was also performed for the macrophages around TNN, PDA@TNN, and AgNPs-PDA@TNN *in vivo*. An increase in the Arg-1 protein level **(Figure 7E)** and a decrease in the iNOS protein level **(Figure 7F)** were detected. The results verified the immunomodulatory ability of AgNPs-PDA@TNN, facilitating the M2 macrophage polarization.

## 3. Discussion

In recent decades, the strategies of biomimetic and bioinspired bone materials have emerged as significant approaches for addressing clinical issues. Several physiochemical properties of Ti alloy-based implants, such as corrosion, wear resistance, stiffness, and biocompatibility, must be considered. With the advances in clinical medicine and biomaterials, it is possible to improve the mechanical performance and long-term durability of advanced synthetic materials 72, through the biomimetics and interdisciplinary strategy 73. In this context, the microstructure of bone 74, artificial skin 75, smart patches 76, microneedles 77, and the functional implant coating can be inspired by nature, e.g., leaves, spider silk, and dragonfly. Therefore, the interdisciplinary development of biomimetics and medical materials offers novel prospects for clinical needs.

The loading and controlled release of antibiotics has been one of the most prevalent strategies to prevent periprosthetic infection. However, overuse of antibiotics might induce potential harm, including antibiotic resistance and the transmission of resistance genes 78. In this study, we adopted a bioinspired strategy to fabricate the topography of insect wings on the surface of Ti implants to achieve the mechanobactericidal ability through the mechanical energy resulting from shock waves, shear stress, pressure, agitation, vibration, or cavitation 79. For instance, it has been reported that bacteria are susceptible to mechanical stress, and the bacterial cell membrane can be easily penetrated by the sharp edges of nanoflakes 80. In nature, the topography of insect wings consists of an array of nanoneedles, which can rupture of the bacterial cell membrane, causing the leakage of contents, and eventually killing the bacteria. Our previous study explored the effects of TNN-based implants on oxygen supply, thereby improving the bactericidal activity of neutrophils in the early stage of infection 20. In this study, we loaded TNN with AgNPs to enhance the mechanobactericidal ability. Further, we examined their effects on macrophages, which play a crucial role in the osteoimmune microenvironment and determine bone regeneration.

As an inorganic antibacterial agent, Ag-based materials display a broad antibacterial spectrum and have been extensively utilized in the clinic 81. Nevertheless, the potential risks of AgNPs, such as decreased cell viability, impaired cell function, and bacterial resistance, have limited their application as implant coatings 82. Our previous study verified that AgNPs within appropriate concentration windows exhibited great biosafety and immunomodulatory ability 15. Considering that the bacterial viability on the surface of bioinspired TNN was significantly reduced, a balance of strong bactericidal ability, biosafety, and immunomodulatory ability can be achieved by introducing a low dosage of AgNPs. Therefore, the mussel-inspired tissue adhesive PDA was used to control the release and alleviate the cytotoxicity of AgNPs.

PDA mitigates the adverse effects of AgNPs in three significant ways. First, PDA is a synthetic analog of melanin, a naturally existing polymer, showing inherent biocompatibility and improving the biosafety of biomaterials 83. Second, the abundant reactive catechol and amine groups of PDA confer adherent ability, achieving the long-lasting release control of the incorporated AgNPs to avoid burst release and reduce cytotoxicity 84. Third, as the final oxidation product of catecholamines, PDA possesses redox properties of catechol and shows excellent ability in transferring electrons and scavenging ROS 85. Therefore, PDA can eliminate the oxidative stress caused by AgNPs.

The synthesis of the PDA layer was empirically based on our previous studies 14, 15, 23. In the present study, we focused on AgNPs rather than PDA. Considering that the synthetic method may also change the thickness of the PDA layer 86, thus affecting the biological impact, fine-tuning the ratio between PDA and AgNPs will be our next research focus.

Osteoimmunology has recently emerged as a novel field, and the close overlap and communication between the immune system and bone metabolism has been recognized 27. With the rapid advancement of osteoimmunology, the early focus on bioengineering has shifted to the design and fabrication of immunomodulatory bone implants 32. In this study, AgNPs-PDA@TNN, a designed bone implant coating, demonstrated the immunomodulatory effect of polarizing the macrophages and avoiding fibrosis encapsulation caused by the foreign body reaction 8. In the osteomicroenvironment surrounding the implant, the manipulated macrophages secrete various cytokines, growth factors, and chemokines to affect signaling pathways and communicate with BMSCs. Consequently, the osteomicroenvironment can positively or negatively promote bone regeneration 87.

During orthopedic surgery, ischemic injury and ischemia-reperfusion increase oxidative stress around the implant site 88. The increased oxidative stress 89 and the inflammatory molecules resulting from the foreign body reaction 90 increase the M1/M2 ratio. However, the enriched phenol groups of PDA can act as radical scavengers, limiting ROS generation and suppressing inflammation in the microenvironment 91. PDA-based biomaterials, including PDA NPs 92, substrates 93, and patches 94, are widely utilized to facilitate M2 polarization, reduce M1 polarization, and create an anti-inflammatory microenvironment for wound healing. Our results showed that PDA@TNN significantly reduced the expression of M1 macrophage markers and increased M2 macrophage markers. Consequently, AgNPs-PDA@TNN could initiate osteogenesis of BMSCs and promote matrix deposition and osteointegration.

Many aspects must be considered in bioengineering to design and fabricate antibacterial implants. The antibacterial strategy should avoid generating drug-resistant bacteria 95 and must consider focusing on clinical translation. In this study, we adopted a bioinspired strategy to fabricate a multi-functionally disordered TNN coating, which demonstrated efficacy in preventing periprosthetic infection and achieved a positive modulation in the osteoimmune microenvironment, avoiding the foreign body reaction and promoting osteointegration.

## 4. Conclusion

We employed the hydrothermal method to fabricate the disordered titanium dioxide nanoneedle (TNN) coating on the surface of pure-Ti and loaded it with silver nanoparticles (AgNPs) and poly-dopamine (PDA). Compared with pure-Ti, AgNPs-PDA@TNN displayed enhanced hydrophilic properties and roughness. The biocompatibility of AgNPs-PDA@TNN was investigated using various assays, which indicated that the PDA layer strongly reduced the cytotoxicity caused by the burst release of Ag^+^, thus reaching a balance between the bactericidal ability and biocompatibility. AgNPs-PDA@TNN also exhibited an excellent immunomodulatory ability, which could notably promote the M2 macrophage and inhibit M1 macrophage polarization. Furthermore, AgNPs-PDA@TNN significantly facilitated osteogenic differentiation of BMSCs and enhanced osteointegration between the implant and the bone. Lastly, this study established the tibial osteomyelitis model in SD rats. Micro-CT scanning and histological analyses verified the outstanding performance of AgNPs-PDA@TNN in preventing periprosthetic infection and promoting bone healing and osteointegration. In summary, AgNPs-PDA@TNN represents a promising surface modification strategy for preventing osteomyelitis, modulating the osteoimmune microenvironment, and enhancing osteointegration.

## 5. Methods

### 5.1 Specimen fabrication

Pure-Ti foil (purity: 99.8%, size: 10 mm×10 mm×1 mm) and pure-Ti rod (1 mm in diameter, 10 mm in length) were used for the specimen preparation. Ti foil and rods were polished, degreased, and dried in nitrogen. Subsequently, the specimens were placed in a Teflon-lined autoclave (temperature 200°C) with 1 M NaOH for 10 h to fabricate sodium titanate (Na2Ti3O7) nanoneedles. The surface of specimens was washed ultrasonically and incubated in HCl for 1h. Then, the specimens were washed with deionized water and dried. Lastly, the TiO_2_ nanoneedles (TNN) were exposed to 400°C annealing temperature for 2h. TNN were immersed in the dopamine hydrochloride (1g/L, Cat No.H8502, Merck KGaA, Darmstadt, Germany)- containing Tris-buffer (10 mM, Cat No.93352, Merck KGaA, Darmstadt, Germany) for 6 h to fabricate PDA@TNN. To load AgNPs on the surface of TNN or PDA@TNN, specimens were immersed in AgNO_3_ solution (100 mM, Cat No.10220, Merck KGaA, Darmstadt, Germany) for 15 min and exposed to ultraviolet light for 30 min to generate AgNPs@TNN or AgNPs-PDA@TNN.

### 5.2 Surface characterization

The surface morphology was observed with scanning electron microscopy (SEM, Cat No.EVO 10, ZEISS, Germany) with 2×10^4^ V. The hydrophilicity of the specimen was evaluated with the water contact assay (JY-82C, Chengde Dingsheng, China). The chemical composition and elemental analysis were performed with X-ray photoelectron spectroscopy (XPS, Thermo Scientific K-Alpha, USA). The surface characterization and roughness were assessed with an atomic force microscope (AFM, Bruker Dimension Icon, Germany). The Ag^+^ release was performed by immersing specimens in deionized water for 1, 3, 5, 7, 10, and 14 days, which was changed daily. The concentration of Ag^+^ was evaluated with the inductively coupled plasma mass spectrometry (ICP-MS, Agilent 5110, USA).

### 5.3 Cell culture

Bone-derived mesenchymal stromal cells (BMSCs) and the macrophage cell line (RAW 264.7) were used in this study. BMSCs were cultured in α-MEM medium (Cat No.12561056, Gibco, USA) supplemented with 10% fetal bovine serum (FBS, Cat No.16010159, Gibco, USA) in a water-saturated atmosphere of 5% CO_2_ at 37°C. For osteogenic differentiation, BMSCs were cultured in an osteogenic differentiation medium (α-MEM medium supplemented with 1% FBS, 200 µM L-ascorbate-2-phosphate, 5 mM β-glycerol-phosphate, 25 mM HEPES, 1.5 mM CaCl_2_, and 100 nM dexamethasone) to 90% confluency. RAW 264.7 cells were cultured in a high glucose DMEM medium (Cat No.11965092, Gibco, USA) supplemented with 10% FBS in a water-saturated atmosphere of 5% CO_2_ at 37°C. The culture medium was changed twice a week**.**

### 5.4 Cell viability

Cell viability was evaluated with the cell counting kit (CCK-8, Cat No.C0038, Beyotime Biotechnology, China). BMSCs were cultured for 1, 4, and 7 days, and RAW 264.7 cells were cultured for 1, 3, and 5 days. The cells were washed 3 times with PBS and incubated with CCK-8 working solution for 1 h at 37°C. Subsequently, the OD value was measured with a microplate reader (PerkinElmer, USA) at 450 nm.

### 5.5 Live/dead staining and cytoskeletal staining

Live/dead staining was performed to evaluate the cytotoxicity and bactericidal ability of the specimens. BMSCs, RAW 264.7 cells, and the adhesive bacteria on the surface of specimens were gently washed 3 times with PBS and incubated with Calcein-AM and PI staining dye of the live/dead viability/cytotoxicity kit (Cat No. L3224, Thermo Fisher Scientific Inc., Shanghai, China) in the plain medium at 37°C for 1 h. The fluorescent images were acquired and documented.

### 5.6 Cytoskeletal staining

To assess the cell adhesion and cytoskeleton alternation, BMSCs and RAW 264.7 cells were washed 3 times with PBS, then fixed with 4% formaldehyde at room temperature for 10 min. The actin cytoskeleton was stained with Actin-Tracker Green-488 (1:100, Cat No.C2201S, Beyotime Biotechnology, China) for 2 h at 37°C, and nuclei were stained with DAPI (5mg/ml, Cat No.C1005, Beyotime Biotechnology, China) for 30 min at 37°C. After gently washing 3 times with PBS, the fluorescent images were taken with a fluorescence microscope (CLSM, Nikon, Japan). Cell spreading was quantified by the cytoplasmic-nuclear ratio.

### 5.7 Immunofluorescence staining

RAW 264.7 cells were fixed in 4% formaldehyde for 10 min at room temperature, then incubated with 0.2% Triton-X-100 for 10 min, and subsequently fixed with 2% formaldehyde for 10 min. Subsequently, cells were blocked with 5% bovine serum albumin (BSA, Cat No.PS113, Epizyme Biotech, China) at room temperature for 1 h. Then, the cells were incubated with primary antibody against Arg-1 (1:5000, Cat No16001-1-AP from Proteintech, China), NOS2 (1:2000, Cat No.22226-1-AP from Proteintech, China), IL-10 (1:1000, Cat No. HA722032 from HUABIO, China), and CD86 (1:5000, Cat No. 26903-1-AP from Proteintech, China) overnight at 4 °C. On the second day, RAW 264.7 cells were washed with PBS and incubated with fluorescent secondary antibody for 2 h and DAPI (5mg/ml, Cat No.C1005, Beyotime Biotechnology, China) for 30 min. Fluorescent images were taken with a fluorescence microscope and analyzed using ImageJ software.

### 5.8 Antibacterial assay

Both gram-positive *S. aureus* and gram-negative *E. coli* were used for the antibacterial assay. The bacterial suspension was seeded in Luria-Bertani (LB) culture medium (Cat No. L1010, Solarbio, China) and cultured for 24 h at 37 °C with shaking. The bacterial suspension was diluted to the concentration of 1×10^5^ CFUs/mL, and 100 μL or 10 μL bacterial suspension was spread on LB agar plates (Cat No. L1015, Solarbio, China), and cultured at 37 °C for 24 h. The images of the agar were then recorded to analyze anti-biofilm and antibacterial ability.

### 5.9 DCFH-DA assay

The production of intracellular ROS was assessed with a 2′, 7′-dichlorofluorescein diacetate (DCFH-DA, Cat No. D6883, Merck KGaA, Darmstadt, Germany) assay. RAW 264.7 cells were washed 3 times with PBS, then incubated with 10 μM DCFH-DA probes at 37 °C in the dark. After 30 min incubation, cells were rewashed, and fluorescent images were taken with a fluorescence microscope (CLSM, Nikon, Japan).

### 5.10 RNA isolation and RT-qPCR

Total RNA of RAW 264.7 and BMSCs was isolated with Eastep® Super Total RNA Extraction Kit (Cat No.LS1040, Promega Biotech Co.,Ltd, Beijing, China). The collected RNA pellets were resuspended in diethylpyrocarbonate (DEPC) water. The concentration and purity of the RNAs were determined with NanoDrop™ (Cat No.840-317400, Thermo Fisher Scientific Inc., Shanghai, China). Subsequently, the total RNA was converted into cDNA with a cDNA synthesis kit (Cat No. D7190S, Beyotime Biotechnology, China). Lastly, the qRT-PCR was conducted using a SYBR Green One-Step RT-qPCR Kit (Cat No. D7268S, Beyotime Biotechnology, China). RT-qPCR was performed according to the manufacturer's instructions. The relative gene expression was calculated using the 2^-∆∆CT^ method, and GAPDH was used as the housekeeping gene. The primers used in this study are summarized in Table 1.

### 5.11 Western blotting

To harvest total protein, BMSCs were lysed in RIPA buffer (Cat No. P0013B, Beyotime Biotechnology, China), and the lysate was centrifuged (3000× g, 10 min) to remove cell debris. The protein concentration was measured by the BCA Protein Assay Kit (Cat No. P0012S, Beyotime Biotechnology, China); 25 µg of total protein were separated by SDS-PAGE (10% acrylamide-bisacrylamide gels, 100 V, 180 min) and subsequently transferred to PVDF membranes (400 mA, 30 min). Protein-Free Blocking Buffer for Western Blot (Cat No. P0240-100ml, Beyotime Biotechnology, China) was used to block the unspecific binding sites.

Membranes were incubated with primary antibodies against BMP-2 (1:1000, Cat No. 66383-1-Ig from Proteintech, China), RUNX2 (1:5000, Cat No. ET1612-47 from HUABIO, China), and SPP1 (1:1000, Cat No. 22952-1-AP from Proteintech, China), and GAPDH (1:10,000, Cat No. ET1601-4 from HUABIO, China), overnight at 4 °C. Subsequently, membranes were incubated with corresponding HRP-labeled secondary antibodies (1:10,000 in TBST) for 2 h at room temperature. Membranes were covered with Omni-ECL™ chemiluminescent substrate solution (Cat No. SQ202, Epizyme Biotech, China), and a CCD camera was used to detect the chemiluminescent signals. Signal intensities were evaluated using the ImageJ software.

### 5.12 Cytokine array

The Proteome Profiler Mouse Cytokine Array Kit (Cat No. ARY006, Bio-Techne, USA) was used to characterize cytokine secretion by RAW 264.7 cells. Briefly, RAW 264.7 cells (5×10^4^ cells/well) were seeded on different specimens in 24-well plates and cultured for 24 h at 37 °C (5% CO_2_, humidified atmosphere). Subsequently, the media was collected and centrifuged (800×g, 10 min) to remove the cell debris. The cytokine array was conducted according to the manufacturer's instructions. The results were quantified using the ImageJ software. The data were normalized to the internal controls and analyzed with the GraphPad Prism software V.8.0.1 (El Camino Real, USA) 96.

### 5.13 ALP staining and Alizarin red staining

ALP and Alizarin red staining were conducted to assess osteogenic differentiation in the early differentiation stage and matrix mineralization in the late differentiation stage. For ALP staining, 2 weeks of osteogenic differentiation was performed. BMSCs were washed 3 times with PBS and incubated with the BCIP/NBT Alkaline Phosphatase Color Development Kit (Cat No.C3206, Beyotime Biotechnology, China). For Alizarin red staining, 4 weeks of osteogenic differentiation was conducted, and BMSCs were subsequently fixed with ice-cold 99% ethanol at -20 °C for 1 h. Afterward, BMSCs were gently washed 3 times with tap water. Then, BMSCs were incubated with Alizarin red solution (Cat No.C0138, Beyotime Biotechnology, China) for 30 min at room temperature. The cells were subsequently washed 3 times with tap water to remove unbound Alizarin red staining dye. The images were recorded with a microscope (CLSM, Nikon, Japan).

### 5.14 Surgical implantation of animal study

All surgical procedures were performed following Sichuan University Guidelines and were approved by the Ethics Committee for Animal Experiments of Sichuan University (20240122008). Sprague Dawley rats (male, 6 weeks old, average weight: 150 g) were obtained from Dashuo Laboratory Animal Co., Ltd., China. A tibial osteomyelitis model was established in rats. All SD rats were randomly divided into 5 experimental groups according to the implantation: pure-Ti, TNN, PDA@TNN, AgNPs@TNN, and AgNPs-PDA@TNN. After anesthesia, the surgical field of the rats was shaved and disinfected, and then an incision was made along the knee joint to expose the proximal metaphysis of the tibia. An electric drill was used to create a channel from the tibial plateau to the bone marrow cavity. Subsequently, the specimens pre-coated with bacteria were implanted into the tibia, muscle, fascias, and skin were carefully sutured. The health conditions of rats, including body weight, temperature, and activity, were monitored daily.

### 5.15 Micro-CT evaluation

Micro-CT (Quantum&nbsp; GX, PerkinElmer, Shanghai, China), 70 kV, and a current of 200 µA, were used to assess the osteomyelitis and osteointegration. All samples were scanned with the micro-CT, and the coronal CT images, 3D reconstruction, and quantitative analysis were generated and documented with the analysis software.

### 5.16 Histological analyses

All samples were first decalcified with 10% EDTA for 30 days, embedded in paraffin and cut into 10 mm sections. The H&E, Masson, Grams, immunohistochemical, and immunofluorescence staining were performed for histopathological analyses. The images were documented with a fluorescence microscope (CLSM, Nikon, Japan).

### 5.17 Statistical analysis

The number of biological (N), and technical (n) replicates for all experiments are provided in the figure legends. The error bars represent the standard error of the mean. One-way analysis of variance (ANOVA) with post-hoc Tukey test was used to determine statistical significance. Differences were considered statistically significant at p < 0.05 (* p < 0.05, ** p < 0.01, *** p < 0.001 and **** p < 0.0001). Statistical analyses were performed using GraphPad Prism software V.8.0.1 (El Camino Real, USA).

## Figures and Tables

**Scheme 1:**
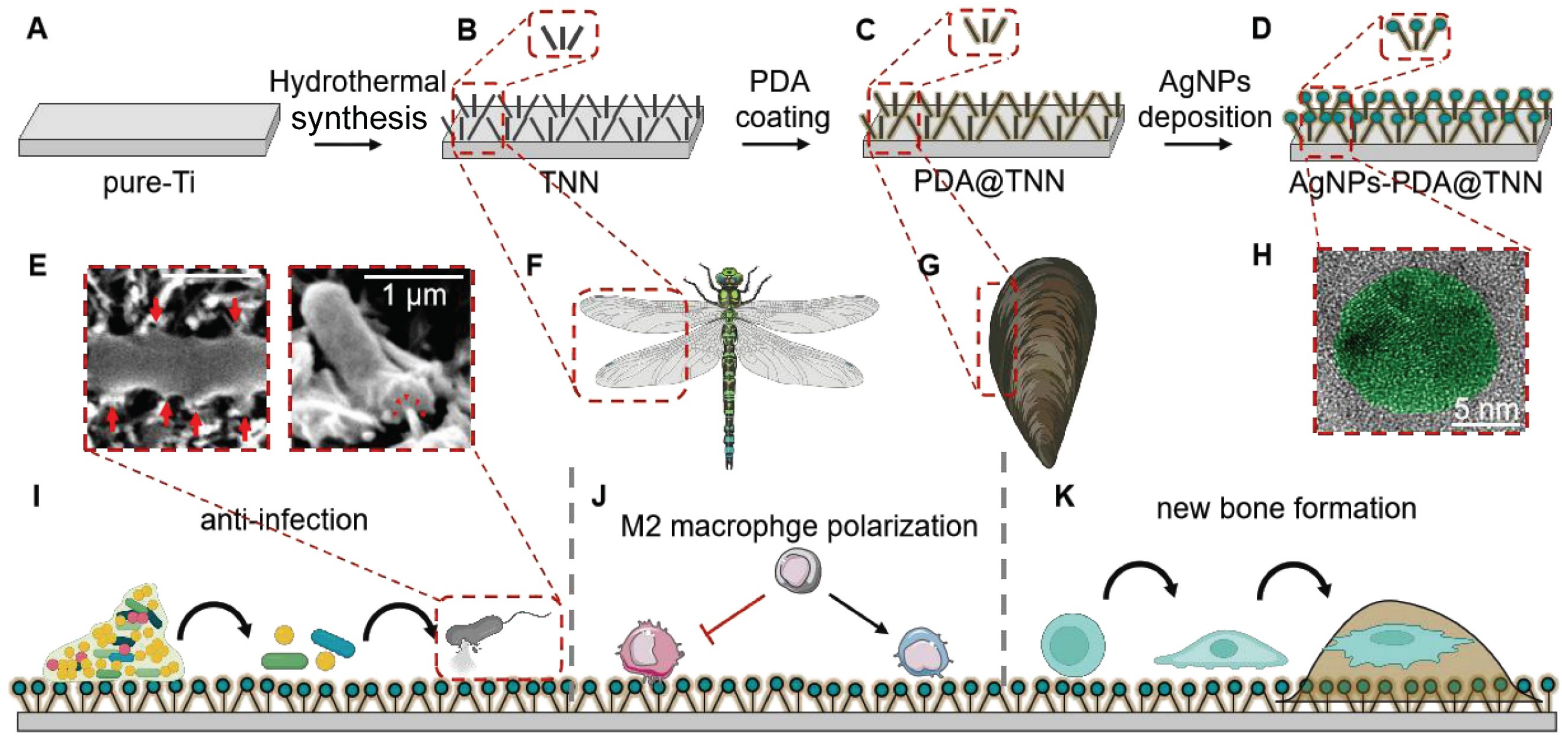
** Illustration of TiO2 Nanoneedles. (A-D)** Synthesis of AgNPs-PDA@TNN: **(A)** Commercial Ti substrate with 99.8% purity was polished, degreased, and dried in nitrogen gas. The pure-Ti substrate was used as the control group or the precursor material of other specimens; **(B)** Subsequently, the hydrothermal synthesis was used to treat the pure-Ti substrate to construct the disordered TNN surface morphology; **(C)** TNN specimen was then immersed in the PDA solution to obtain the PDA coating on the surface of TNN; **(D)** Finally, the PDA@TNN specimen was immersed in the AgNO_3_ solution and exposed to UV light to facilitate the AgNPs deposition on the surface of PDA@TNN and fabricate the AgNPs-PDA@TNN. **(E)** The disordered TNN surface can mechanically stretch and puncture the bacterial wall. Consequently, the bacteria would be killed due to the disruption of the bacterial wall andleakage of intracellular contents. **(F)** The nanostructure of the TNN coating was inspired by the topography of insect wings, which exhibit remarkable bactericidal ability in nature. **(G)** The PDA coating was inspired by the mussel adhesion protein, which can improve biocompatibility and confer the implant with release control capability. **(H)** SEM image of the AgNP coated on the surface of the specimen. The design and fabrication of the multi-functional coating in this study aimed to **(I)** Disrupt the biofilm on the implant, puncture the bacterial cell wall, cause leakage of the intracellular contents of the adhesive bacteria, and kill the planktonic bacteria through the long-lasting release of AgNPs. **(J)** Modulate the local immune microenvironment by facilitating M2 macrophage polarization, which resulted in an osteoimmune microenvironment. **(K)** The ultimate therapeutic goal of the multi-functionally coating in this study was to support the new bone formation and achieve favorable osteointegration.

**Table 1:** Primers used in RT-qPCR

Gene	Accession Number	Forward Primer (5'-3')	Reverse Primer (5'-3')
*mNOS2*	NM_010927	GTTCTCAGCCCAACAATACAAGA	GTGGACGGGTCGATGTCAC
*mTNFα*	NM_013693	CAGGCGGTGCCTATGTCTC	CGATCACCCCGAAGTTCAGTAG
*mCD86*	NM_019388	TCAATGGGACTGCATATCTGCC	GCCAAAATACTACCAGCTCACT
*mArg-1*	NM_007482	CTCCAAGCCAAAGTCCTTAGAG	GGAGCTGTCATTAGGGACATCA
*mIL-10*	NM_010548	CTTACTGACTGGCATGAGGATCA	GCAGCTCTAGGAGCATGTGG
*mMrc-1*	NM_008625	CTCTGTTCAGCTATTGGACGC	TGGCACTCCCAAACATAATTTGA
*mITGα5*	*NM_008402*	CGGGTCCCGAGGGAAGTTA	TGGATGAGCATTCACATTTGAGA
*mITGβ3*	NM_016780	GGCGTTGTTGTTGGAGAGTC	CTTCAGGTTACATCGGGGTGA
*mRUNX2*	NM_001146038	GACTGTGGTTACCGTCATGGC	ACTTGGTTTTTCATAACAGCGGA
*mCol1A1*	NM_007742	GCTCCTCTTAGGGGCCACT	ATTGGGGACCCTTAGGCCAT
*mSPP1*	NM_001204203	ATCTCACCATTCGGATGAGTCT	TGTAGGGACGATTGGAGTGAAA
*mIBSP*	NM_008318	ATGGAGACGGCGATAGTTCC	CTAGCTGTTACACCCGAGAGT
*mBMP2*	NM_080708	GGCCGAAGGTGGATTCTCC	GTCGGGTGTGTTATTGACATACA
*mBMP4*	NM_007554	ATTCCTGGTAACCGAATGCTG	CCGGTCTCAGGTATCAAACTAGC
*mGAPDH*	NM_008084	AGGTCGGTGTGAACGGATTTG	GGGGTCGTTGATGGCAACA
